# Hydroponic lettuce *in-situ* water circulation evaluation via nondestructive mass measurement in controlled environment

**DOI:** 10.3389/fpls.2024.1385191

**Published:** 2024-10-16

**Authors:** Yanhua Huang, Zheng Ni, Yanbin Chang, Lizhi Wang

**Affiliations:** ^1^ Department of Industrial and Manufacturing Systems Engineering, Iowa State University, Ames, IA, United States; ^2^ Innovation Science and Engineering Lab, Bayer Crop Science, St. Louis, MO, United States; ^3^ School of Industrial Engineering and Management, Oklahoma State University, Stillwater, OK, United States

**Keywords:** IoT, hydroponic lettuce, water circulation, non-destructive evaluation, *in situ*, transpiration, environmental susceptibility, operation optimization

## Abstract

This study proposed a hydroponic system with the capacity to acquire high-resolution *in situ* mass data for non-destructive evaluation of water circulation in lettuce. The system customizes the watering profile, enables high-frequency *in situ* weight measurement, and monitors multidimensional environment changes. Key air, water, and light parameters were collected to evaluate the plant response, susceptibility, and adaptability to environmental conditions. Multiple physiological indices were defined to characterize the properties of two lettuce varieties in response to different environmental factors.

## 1 Introduction

Water circulation plays a crucial role in major plant physiological processes, such as respiration and photosynthesis. In respiration, water movement affects oxygen availability, root growth and health, and nutrient uptake. Mittler et al. (2022) reviewed important oxygen-induced signaling in the plant respiration process, where most of the oxygen in hydroponic plant access is from circulated water. Bharti et al. (2019) discovered that changed water conditions increase root mass weight by 27%–40% and also improve nitrogen (N) absorption rate. In photosynthesis, water plays a more important role in nutrient transportation, chemical reactions, carbon dioxide (CO_2_) capture, oxygen production, and adenosine triphosphate (ATP) production (Papanatsiou et al., 2019; Sun et al., 2017; Woodward, 2004). As one of the key carbon sources in photosynthesis, along with water, chlorophyll combines them to produce ATP and oxygen (Sun et al., 2017).

The objective of this study is to design an Internet of Things (IoT) integrated system in a hydroponic environment for collecting data of water circulation throughout the plant growth process, which could be used to estimate the physiological properties of plants. IoT can be a very useful technology for collecting data in controlled environments such as hydroponic systems. Typical IoT hydroponic system integration includes sensor technology, data collection and communication, automation and control, decision support systems, energy optimization, remote monitoring and troubleshooting, scalability and integration, and data-driven insights and optimization. Mishra et al. (2020) used sensor arrays to improve the accuracy of nutrient concentration measurement in hydroponic systems to optimize environmental conditions for plants. Ibayashi et al. (2016) improved the stability and reliability of data communication for tomato hydroponics on a locally deployed wireless control system. Saraswathi et al. (2018) designed an IoT system to monitor and control the nutrients, pH, electrical conductivity, air temperature, and humidity via internet data communication and mobile applications. Optimal operation prescriptions can be recommended by crop modeling (CM) and executed by the controlling system, which closes the loop of measurement, calibration, and justifications (Gallardo et al., 2020). Integration of IoT’s control capability and crop models’ physiological insights also supports research in plant genotype identification and isolation (Elsallam et al., 2021) and regulation of energy consumption (Elsallam et al., 2021).

A major challenge in understanding the role of water circulation in the crop growth process is to observe and interpret water movement under different environmental conditions. Water uptake and loss change plant weight significantly. It is necessary to continuously track water movement at different environmental conditions to correlate the water intake and loss to environmental conditions, providing direct experimental evidence of how the environment alters plant respiration and photosynthesis. Therefore, in order to study water circulation in the plant growth process in a hydroponic environment, three categories of data must be collected: air, water, and light.

- First, air data include air temperature, humidity, and concentration of carbon dioxide. Air temperature impacts both the photosynthesis and respiration processes that fundamentally regulate plant growth activities, including water transpiration, stomata open percentage, carbon dioxide absorption rate, cellulose synthesis rate, and cell replication rate (Zhang et al., 2022). Humidity controls the stomata open percentage water transpiration potential between leaf and root (Chia and Lim, 2022). Carbon dioxide (CO_2_), as the major carbon source in the photosynthesis process, determines the growth rate of plants (Gamage et al., 2018).
- Second, water data include water temperature, electric conductivity, and pH. Watering temperature, also reported as root zone temperature, has been shown to affect lettuce yield. Thakulla et al. (2021) discovered that lettuce gained the highest fresh and dry shoot weight at 21.1°C and generated most sugar at 18.3°C. Electric conductivity indicates the nutrient concentration in the circulating media. Neocleous and Savvas (2022) attempted to optimize electric conductivity for smart nutrient solutions that feed hydroponic crops. Controlling water pH mainly aims for more balanced nutrient ions’ availability. Most alkaline earth metal ions, such as calcium (Ca) and magnesium (Mg), do not precipitate at acidic levels when plants can utilize them in the water circulation (Sambo et al., 2019).
- Third, light data include light intensity and photoperiod. When exposed to different spectrums of light, plants take nutrition differently. Ohashi-Kaneko et al. (2006) reported that nitrogen utilization was different when plants were or were not exposed to blue light as a supplement for red light radiation. Changing photoperiods also modifies plant growth. Samuolienė et al. (2021) discovered that 16-h-per-day photoperiods are optimal for lettuce (*Lactuca sativa* L, “Lobjoits Green Cos”), synthesizing the highest xanthophylls with red and blue light exposure.

In this study, we designed and constructed a non-destructive IoT-integrated hydroponic cultivation system with the capability to monitor and control air, water, and light parameters. Correlation maps were calculated for plant growth analysis based on 10 environmental parameters and 14 evaluation indices describing physiological and operational efficiencies extracted from weight data signals. Insights from our analysis could be used by hydroponic growers and researchers to estimate parameters in crop models, interpret observations of the respiration and photosynthesis processes in the hydroponic cultivation system, predict crop yields, improve quality (such as taste, nutritional value, and shelf life), increase crop yield, and reduce energy consumption.

The goal of the proposed hydroponic system was not to produce good lettuce; rather, it was to produce good data (especially weight data) to help understand the physiological properties of two lettuce varieties. As a result, this study will not only help identify better environmental conditions for lettuce cultivation but also inspire the design of similar systems to benefit the research and development in other crops.

## 2 System construction and management

**Figure 1:**
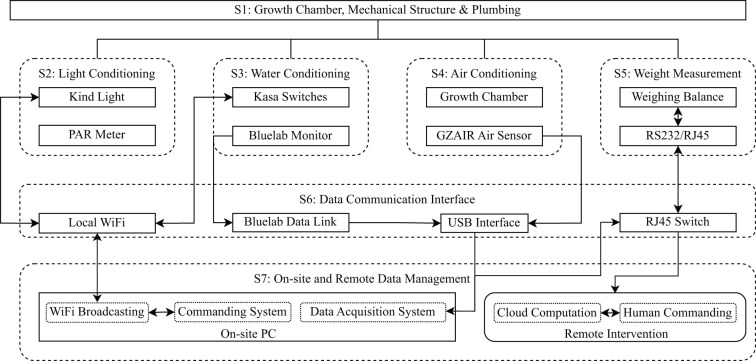
System architect and data flow. All rectangle boxes are physical components, all round-corner boxes are subsystems, all solid boarded boxes are physical hardware, and all dashed boxes are functional components. Arrows indicate the data flow directions.

We designed a hydroponic system for growing Romaine and Oak Leaf lettuce (*Lactuca sativa*), which features real-time, high-frequency (1 Hz), and high-resolution (0.01 g) mass data acquisition and environmental control for air, water, and light. The system consists of seven subsystems, namely, S1: Mechanical structure and plumbing, S2: Air, S3: Water, S4: Light, S5: Weight measurement, S6: Data communication interface, and S7: On-site and remote data management. The system structure is shown in **Figure 1** and details are explained in subsequent sections.

### 2.1 S1: Mechanical structures and plumbing

Four independent and identical systems residing in four Percival (Perry, IA) growth chambers were used to facilitate the air temperature and relative humidity monitoring and control.

**Figure 2:**
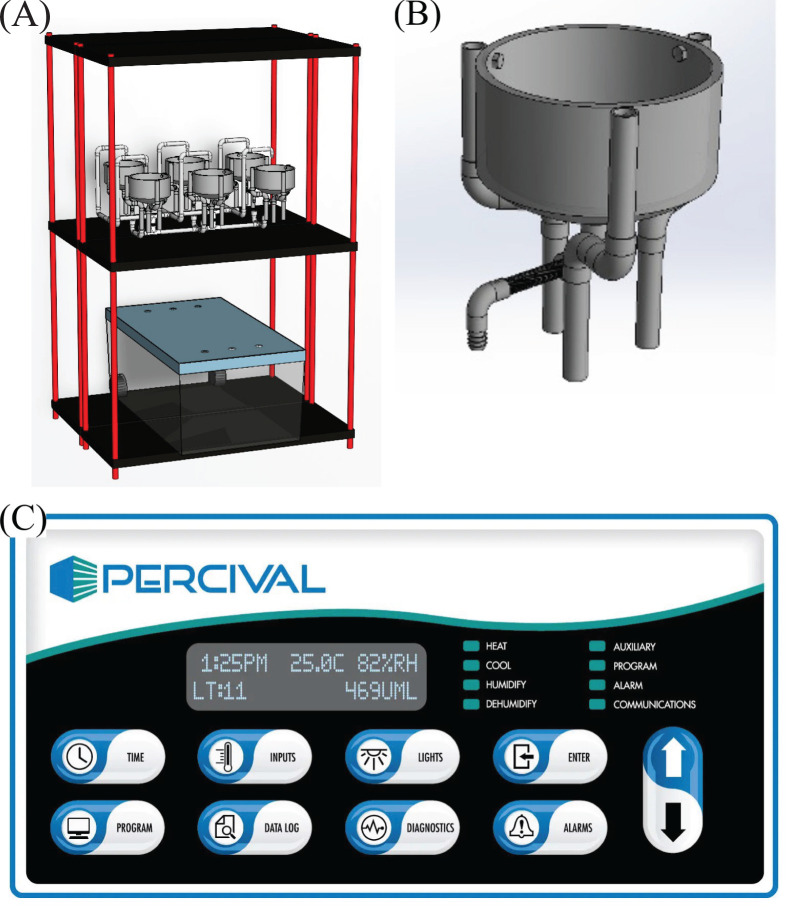
Base level system hardware: growth chamber, mechanical structure, and plumbing system. **(A)** Shelving and plumbing overlook; **(B)** incubating pot assembly ensures no water residue after water exits; **(C)** air temperature and relative humidity control access point.

To manage water delivery in detail, a complex water management system is designed and optimized to fit chamber and shelf setup, as illustrated in **Figure 2A**. Each incubation chamber contains two units, presented in **Figure 2A**. The unit contains a three-layer shelf, a 120-L plastic container for water storage, a water upstream pipeline, six incubating pots, and a water downstream pipeline. Water upstream and downstream pipelines and incubating pots are mainly constructed with PVC parts.

The incubating pot was designed and optimized for hydroponically growing Romaine and Oak Leaf lettuce with flowing aqueous nutrient solution, considering easy assembly, water flowability, compatibility with weight balance measurement system, reliability, and flexibility, as illustrated in **Figure 2B**.

For accessibility of the incubating pots, all PVC was purchased directly from local plumbing vendors. Three-quarter-inch pipes were cut down to the designed length for assembly, whereas other parts were used directly as purchased. The three supporting legs can be rotated to fit different sizes of weighing scale trays. A water exit was designed at the bottom of the 6-inch cap to eliminate dead water. Leg tops were adhered to and fastened onto the cap with PVC cement, stainless bolts, and nuts. Such a design took thermal geometry variation, high humidity, and high salty conditions into consideration.

### 2.2 S2: Air conditioning and evaluation subsystem

System air conditions were primarily regulated by a Percival growth chamber mentioned previously via prior defined programs and an on-board interface, as shown in **Figure 2C**. Programs could define desired temperature and relative humidity with customized time within a 24-h cycle. The chamber operation conditions were logged and could be downloaded from an on-chip micro-SD card. A separate air condition data logger, GZAIR CO_2_ Data Logger Monitor (Engerwitzdorf, Austria), was used to log air temperature, humidity, and CO_2_ concentration with an acquisition frequency of once per 5 min. This data logger was connected to an on-site PC via a USB cable.

### 2.3 S3: Water conditioning subsystem

Water conditioning included two parts: scheduling and nutrient conditions. Kasa WiFi-controlled switches (Shenzhen, China) were used to control the Smartpond 80-gallon-per-hour submersible fountain pump purchased from Lowes.com to customize water scheduling. The Flora Nova Grow 7-4-10 one-part nutrient solution, manufactured by General Hydroponics (Berkeley, CA), was diluted 1,600 and 800 times with tap water as two nutrient solutions during the seedling and early growing stages. Complete composition of this formula can be found at https://generalhydroponics.com/resources/floranova-grow-product-label/.

Watering events alter the measured weight significantly. By analyzing measured weight data, watering events were clearly logged and carefully evaluated. Water conditions, including water temperature, pH, and electric conductivity, were monitored by Bluelab (New Zealand) Wi-Fi-enabled guardian monitor.

### 2.4 S4: Light conditioning subsystem

Lighting was provided by WiFi-enabled K5 series XL750 KindLED (Santa Rosa, CA) grow light. This lighting system only enabled individual control of white, red, and blue lights, although other spectrums of light could also affect plant growth. Photosynthetic photon flux density (PPFD), also known as light intensity, could be modified as variations of power percentage, ranging from 0% to 100%. Lighting conditions could be tuned with minute resolution in the time domain.

Since PPFD was controlled digitally (not analog signals), the delivered PPFD measurement was carried out as photosynthetic active radiation (PAR) meter calibration at the incubating pot level. Each light channel was calibrated individually. Light data were based on calibrated PPFD readings and populated with a frequency of minutes on the time domain.

### 2.5 S5: Weight measurement subsystem

The weight of each growing lettuce was continuously recorded using a weight measurement system, which consisted of Ruishan (Shanghai, China) weighing balances and Serial RS232-to-Ethernet converters. The capacity and accuracy of the weighing balance were 2,000 g and 0.01 g, respectively. Since the data port of the weight balance was the RS232 serial port, model number USR-TCP232-302 converters, manufactured by PUSR (Jinan, China), were used to convert serial signals to transmission control protocol/internet protocol (TCP/IP) signals.

### 2.6 S6: Data communication Interface

Four different interfaces were used to communicate sensing and controlling hardware and computation hardware to ensure the stability and reliability of the data communications. With Wi-Fi connections, managerial commands and system status, such as feedback from Kind LED lights and Kasa switches, could communicate back and forth over the Wi-Fi interface. In most cases, chambers were maintained at high humidity levels. A wireless communication approach improved system safety and data communication reliability. All Bluelab monitors were required to upload data via a proprietary Bluelab data link, and a Bluelab connect stick was used to receive wireless data from Bluelab monitors and send it to the on-site PC for data logging via the USB interface.

Besides water condition data, air condition data were also transmitted via the USB interface. Water data were logged with a frequency of 1 min, and air condition data were logged every 5 min. TCP/IP protocol was utilized to transmit high-frequency data such as weight measurement data communication and remote data exchange. T-568B terminated category 6 cables were used to ensure hardware reliability and transmitting speed.

### 2.7 S7: On-site and remote data management

One on-site computer (PC) was set up per growth chamber to serve as a secure Wi-Fi broadcaster, commanding system, and data acquisition system. Beyond three environmental condition subsystems and a weight measurement system, 16 web cameras are implemented for operational status checks, condition visual verification, visual inspections, and remote system failure diagnoses. With the large numbers of devices connected via Wi-Fi, a secure and independent broadcast is required to ensure command executing security and reliability. Since the Kind LED light on-board chip uses a web page-based control interface, this on-site PC is perfect for providing remote access to the lighting system via internet connection. The water condition system, air conditioning system, and weight management system all use this PC as a temporary data storage and hub for remote communication.

Beyond this on-site PC, remote computational interventions were needed to provide cloud computing and human intervention access portals. Crop modeling could be hosted on this remote computation equipment, providing model training and updating with *in situ* data input, operational command suggesting and advising, bolting predictions, and breeding simulations. It also provided remote human intervention access portals, where operation managers could access data and modify operation parameters.

## 3 Design of experiments and evaluation approach

The designs of the experiments emphasized creating variability over the growth cycle to reduce the numbers of plant samples needed for data analysis. In total, 24 days of 11 plants’ environmental data and weight measurement data were collected and processed. No crop was harvested during the experiment, since the purpose of the hydroponic system was to produce good data rather than lettuce. All environmental parameters are listed in **Table 1**. In the following subsections, we introduce air, water, and light conditions during the experiments as well as our new definitions for weight data analysis.

**Table 1:** Environmental parameters.

No.	Definition	Denotation	Unit
1	Air temperature	*T_a_*	Celsius
2	Relative humidity	RH	%
3	CO_2_ concentration	*CO*_2_	ppm
4	Electric conductivity	EC	ppm
5	pH	pH	unitless
6	Water temperature	*Tw*	Celsius
7	White (micro) light PPFD	*PPFD_w_*	*µmol/m^2^/s*
8	Red light PPFD	*PPFD_r_*	*µmol/m^2^/s*
9	Blue light PPFD	*PPFD_b_*	*µmol/m^2^/s*
10	Watering times	*No_wt_*	count

### 3.1 Air conditions

Three daily programs were executed through the seedling and cloning stages to differentiate the conditions, as shown in **Table 2**, where programs #1 and #2 had a 24-h cycle and program #3 had a 12-h cycle. Since air circulation in the growth chamber was regulated via a fan with inconstant working schedules, no CO_2_ generation source was introduced in the system. System air condition data, including air temperature, humidity, and CO_2_ concentration, were logged every 5 min per sensor limitation.

**Table 2:** Incubator program parameters.

Program	#1	#2	#3
Daytime start time	7:00	6:00	6:00 and18:00
Daytime temperature (°C)	20	22	20
Daytime humidity (%)	40	50	50
Nighttime start time	19:00	18:00	12:00 and 00:00
Nighttime temperature (°C)	16.5	18	18
Nighttime humidity (%)	20	30	30

### 3.2 Water conditions

For every six plants, 100 L of diluted nutrient solutions were formulated and stored in the 120-L plastic container for hydroponic circulation. To compensate for evaporation, additional tap water was added to maintain the 100-L total volume. In the seedling stage, the flora nutrient was diluted 1,600 times.

In the cloning stage, the flora nutrient was diluted 800 times. At the start, the water’s reading was 450 ppm (in the ppm 500 scale) for electric conductivity, 19°C for water temperature, and 6.8 for pH. Water conditions were logged with 1 min resolution on time domain.

### 3.3 Light conditions

The LED light was positioned 1 m above the incubating pot, which is shown in **Figure 2B**. The lighting conditions were programmed with power percentage and changing time. In the 24-day project duration, the light programs are prescribed in **Table 3**.

**Table 3:** Light conditions prescription.

Time	Red intensity (%)	Blue intensity (%)	White (micro) intensity (%)
08:45:00 a.m.	20	40	40
09:15:00 a.m.	10	25	25
09:45:00 a.m.	5	5	5
10:00:00 a.m.	0	0	0
04:00:00 p.m.	5	5	5
04:15:00 p.m.	10	25	25
04:45:00 p.m.	20	40	40
05:15:00 p.m.	30	60	60

**Figure 3:**
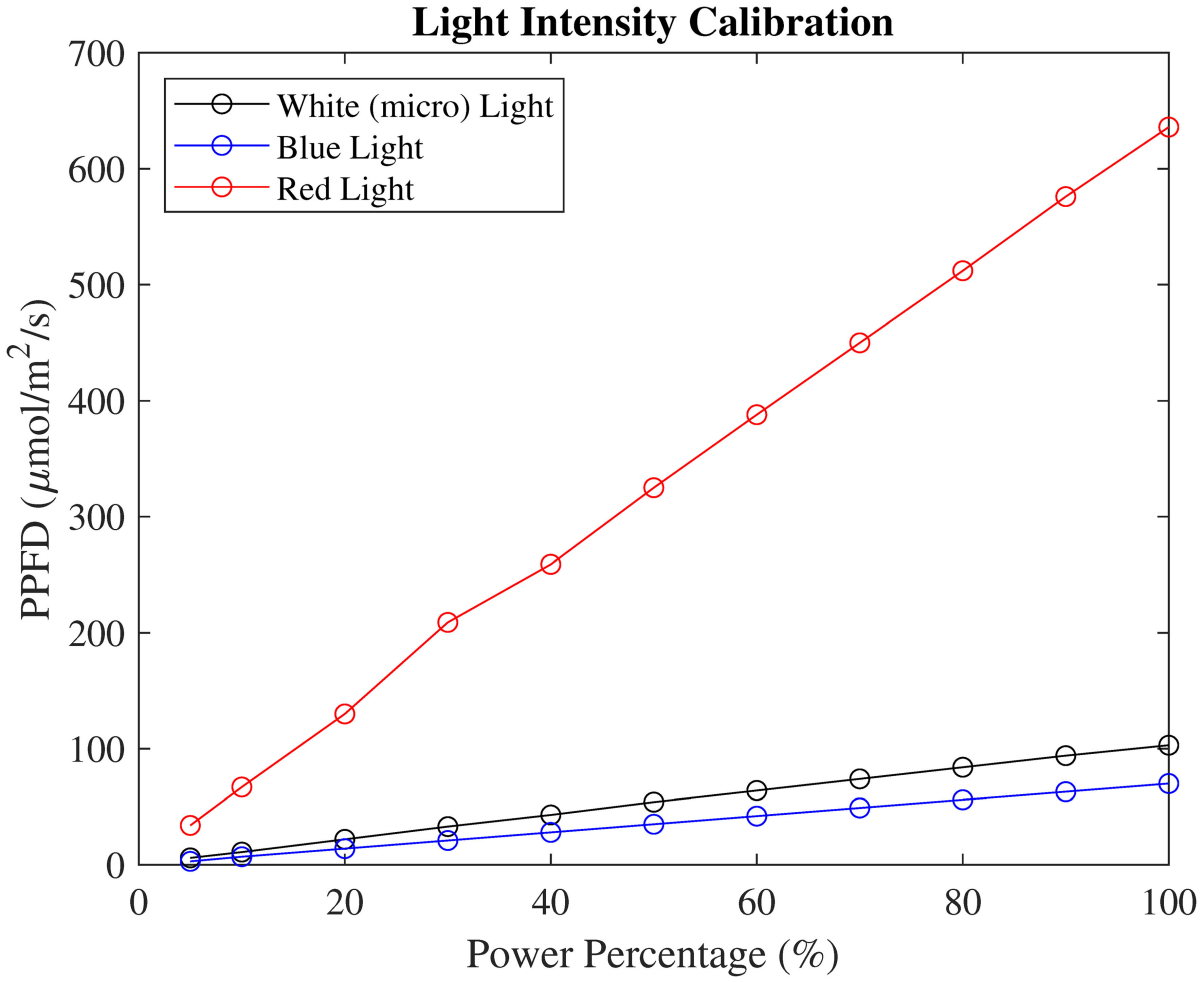
PPFD calibration of white, blue, and red spectrum lights with different light power settings.

For PPFD calibration, all three spectrum lights were calibrated individually. The PAR sensor remained at the same location during calibration and only lighting conditions were changed. The calibration results showed a highly linear correlation with power percentages, as shown in **Figure 3**.

Based on this calibrated PPFD reading, the light condition profiles were populated with minute time resolution, noted as PPFD*_s_* (*t*), where *s* denotes the standard profile. When determining the PPFD for each plant, secondary calibrations were conducted. The PAR sensor was located at each plant’s incubating pot height level. During the calibration, the Kind LED light was set as 60% in red, white, and blue light. The calibrated PPFD for this setting was 494 µmol/m^2^/s. For each plant, a new PPFD reading was logged as ${\text{PPFD}}_{XX.c}$ with XX denoting the plant number. Therefore, the light condition profiles for each plant, ${\text{PPFD}}_{XX}\left(t\right)$ , can be calculated as


(1) $${\text{PPFD}}_{XX}\left(t\right)=\frac{{\text{PPFD}}_{XX.c}}{494}\;\times \;{\text{PPFD}}_{s}\left(t\right)$$

### 3.4 Weight data and physiological indices

**Figure 4:**
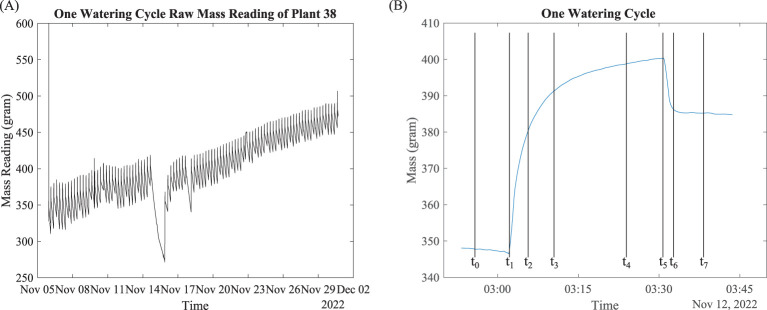
Raw mass data overlook and signal processing. **(A)** Mass data; **(B)** mass data of one watering cycle.

Weight data included weight reading and the corresponding time stamp, as shown in **Figure 4A**. The periodic mass fluctuations were due to the watering event, water evapotranspiration, and root water uptake. To understand the complex effects of multiple processes that could affect the weight data, we make several definitions based on the observed data.

Consider Plant 38 as an example. The mass data of this plant in November 2022 are plotted in **Figure 4A**. **Figure 4B** shows the mass data over a typical watering cycle, where we define eight critical time points:

$t_{0}$ Five minutes before start of watering

$t_{1}$ Start of watering event, when the mass starts to increase rapidly

$t_{2}$ Start of water saturation, when the increase of mass slows down

$t_{3}$ Five minutes after start of water saturation

$t_{4}$ Five minutes before end of watering

$t_{5}$ End of watering event, when the watering stops and mass starts to decrease

$t_{6}$ End of water dripping, when the water loss due to dripping slows down

$t_{7}$ Five minutes after end of water dripping

We define the following physiological indices to characterize plants’ physiological properties and operational effectiveness during the watering cycle:

-
$D_{r}=t_{2}-t_{1}$ , rinsing period
-
$D_{ur}=t_{6}-t_{5}$ , unrinsing period
-
$D_{st}=t_{5}-t_{2}$ , saturation period
-
M(t) , mass of plant at time point t
-
$M_{dy.h}=M\left(t_{2}\right)-M\left(t_{1}\right)$ , dynamic water holding, which is the mass gain during the rinsing period
-
$M_{st.h}=M\left(t_{6}\right)-M\left(t_{1}\right)$ , static water holding, which is the mass gain between the beginning and end of the watering event
-
$M_{dy.l}=M\left(t_{6}\right)-M\left(t_{5}\right)$ , dynamic water loss, which is the mass loss during the unrinsing period
-
$M_{dy.r}=M\left(t_{5}\right)-M\left(t_{2}\right)$ , dynamic root holding, which is the mass gain during the saturation period during root holding of the water
-
$R_{tr.B}=\frac{M\left(t_{1}\right)-M\left(t_{0}\right)}{t_{1}-t_{0}}$ , which is the rate of mass change before the watering event.
-
$R_{ws}=\frac{M_{dy.h}}{t_{2}-t_{1}}$ , which is the rate of mass change during the water rinsing period.
-
$R_{st.B}=\frac{M\left(t_{3}\right)-M\left(t_{2}\right)}{t_{3}-t_{2}}$ , which is the rate of mass change after water saturation.
-
$R_{st.A}=\frac{M\left(t_{5}\right)-M\left(t_{4}\right)}{t_{5}-t_{4}}$ , which is the rate of mass change before watering ends.
-
$R_{we}=\frac{M_{dy.l}}{t_{6}-t_{5}}$ , which is the rate of mass change during the water unrinsing period.
-
$R_{tr.A}=\frac{M\left(t_{7}\right)-M\left(t_{6}\right)}{t_{7}-t_{6}}$ , which is the rate of mass change after water dripping ends.

## 4 Results and discussion

In this section, we present the physiological indices defined in Section 3.4 for plant 38. Data for other plants are available upon request.

### 4.1 Rate evaluation indices

**Figure 5:**
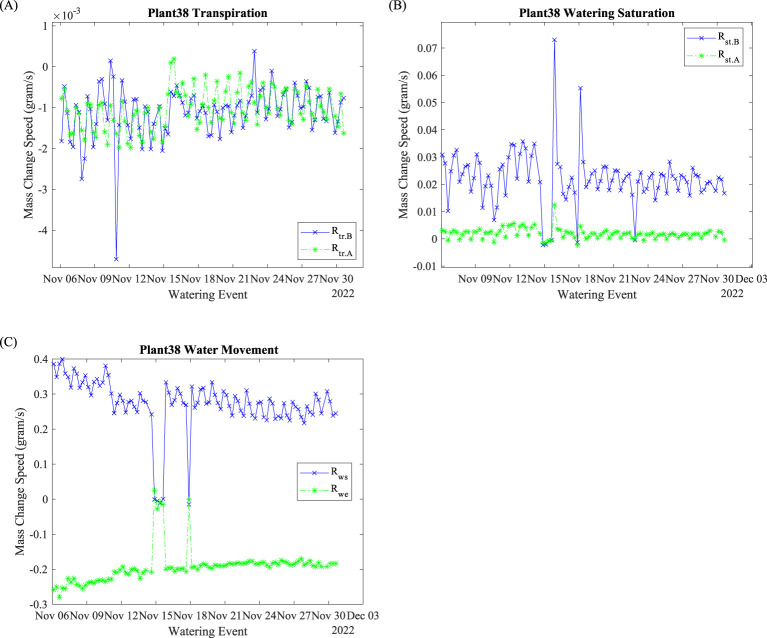
Plant 38: Rate evaluation indices. **(A)** Transpiration rates; **(B)** saturation rates; **(C)** water absorption and exiting rates.

Plant 38’s transpiration rates before and after watering events are shown in **Figure 5A**. The negative data indicate mass loss. Minor positive data points may be caused by balance kickback. Before November 15 and after November 22, pre-watering transpirations were slower (less negative). After the water outage on November 14, the pre-water transpiration was faster (more negative) until November 22. Both pre- and post-transpiration were changing periodically with a cycle of 24 h. Most of the data points ranged from 0 to –2 mg/s.

Watering saturation speeds $R_{st.A}$ and $R_{st.B}$ are shown in **Figure 5B**. In general, $R_{st.B}$ is greater than $R_{st.A}$ . The $R_{st.B}$ had a range from 0.01 to 0.04 g/s, the $R_{st.A}$ ranged from 0 to 0.05 g/s. The periodic (daily) fluctuations reduced as time progressed for both $R_{st.B}$ and $R_{st.A}$ .

The water absorption rate $R_{ws}$ and $R_{we}$ are shown in **Figure 5C**. Before the November 15 water suspension, $R_{ws}$ decreased, and $R_{we}$ increased (less negative) over time. After November 18, both $R_{ws}$ and $R_{we}$ stabilized. They also fluctuated periodically with a 24-h cycle throughout the entire process. The watering absorption speed $R_{ws}$ reflects the plant’s water uptake capability. This capability is determined by xylem osmotic potential as Earles discovered (Earles et al., 2015). Therefore, the $R_{ws}$ can be used as a xylem index to evaluate plant physiology. Water exiting speed, $R_{we}$ , on the other hand indicates the water exit rate. During the growth, plants’ roots were blocking the water exit. The slight increase (less negative, 0.008 g/s/day with *R* ^2^ = 0.75) can be used as an index evaluating root growth conditions.

Environmental factors, such as lighting and air conditions, significantly impact plants’ mass change rate, including $R_{tr.B}$ , $R_{tr.A}$ , $R_{st.B}$ , $R_{st.A}$ , and $R_{ws}$ . As shown in **Figure 5**, most of the mass changing speed fluctuates daily. We suspect that the mass change speeds are correlated with air or lighting conditions since air conditions and light conditions are changed with a 24-h cycle as mentioned in Sections 3.1 and 3.3. On the other hand, the $R_{tr.B}$ and $R_{tr.A}$ behaved differently before the water outage and during the recovery period (November 18 to 21). With uninterrupted watering, plants may utilize sufficient water for respiration to synthesize cellulose and for photosynthesis to accumulate carbon from air. When water availability is limited, plants may modify their photosynthesis and respiration activities to adapt to the water stress physiologically and biochemically (Osakabe et al., 2014).

The watering saturation mass change speed reflects the system watering efficiency. The daily fluctuations indicate that the $R_{st.B}$ is plant physiology related. It signifies the approaching of efficient watering of the plant. Such evaluation indices can be used as an operational trigger to regulate watering time span to optimize the watering event efficiency. The $R_{st.A}$ had less daily fluctuations, compared to $R_{st.B}$ . With universal watering length, the approaching zero of $R_{st.A}$ signifies that watering reached the plant’s dynamic maximum water holding capacity. Since a considerable amount of water will exit the plant after the watering event ends, modifying the watering length to increase $R_{st.A}$ will improve the watering utilization.

### 4.2 Duration evaluation indices

**Figure 6:**
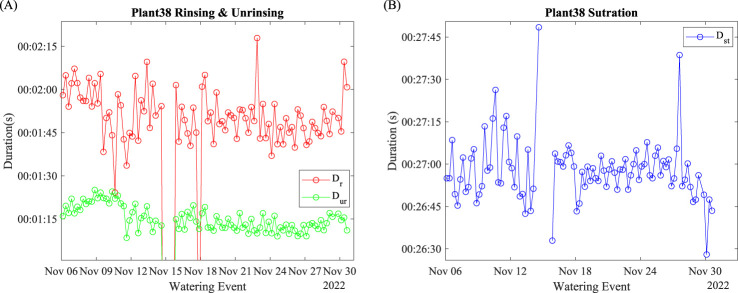
Plant 38: Duration evaluation indices. **(A)** Rinsing and un-rinsing; **(B)** saturation.

The duration of rinsing and un-rinsing of Plant 38 is shown in **Figure 6A**. Abnormal data off the chart were due to watering activity suspension. Regardless of the abnormal data points, the average $D_{r}$ was 104 s, and the average $D_{ur}$ was 72 s. Both $D_{r}$ and $D_{ur}$ fluctuated daily.

The duration of watering saturation is shown in **Figure 6B**. Abnormal data were removed due to watering suspension. Before November 15, the daily fluctuation was larger than after November 18. After November 24, the moving average of the saturation duration decreased significantly.

The duration of rinsing, $D_{r}$ , in **Figure 6A** signifies the time period of no water was wasted after the entire watering event. Since the power of the watering pump was constant, the rising duration indicates the amount of water stress the plant was under at the start point of the watering event. Reduced fluctuations along the growing process indicate that the plant adapted to this watering fashion (amount and frequency). The duration of unrinsing, $D_{ur}$ , in **Figure 6A**, indicates the period of water exiting after the watering event ends. During this period, part of the dynamically held water exited from the incubating pot. Suppose the hydroponic production was carried out because the plant may need to leave the water tank after watering. In that case, the $D_{ur}$ indicates the time needed to water until all the unused nutrient liquid exited the plants.

The duration of saturation, $D_{st}$ , in **Figure 6B**, signifies the period when the plant did not absorb a portion of the pumped water during the watering event. The larger fluctuations before November 15 indicate that daily changed environmental factors significantly impacted the water uptake. The reduced fluctuation after the water outage suggested that the water had already adapted to the current watering fashion, as $D_{r}$ indicates. It may also suggest that the plant was less susceptible to daily cycled environmental parameters. The moving average decrease after November 27 suggests that plants need more time to begin saturation, which indicates the improvement in watering efficiency.

### 4.3 Mass evaluation indices

**Figure 7:**
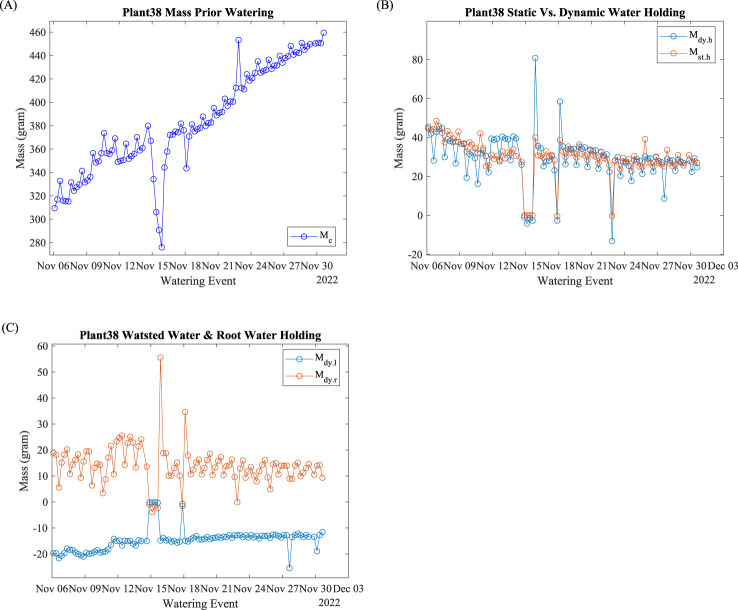
Plant 38: Mass evaluation indices. **(A)** Mass calibration; **(B)** dynamic and static water holding capacity; **(C)** dynamic water loss and root holding capacity.

Mass data at the beginning of the watering event are plotted in **Figure 7A**. This is the lowest mass reading within the watering cycle, which includes the mass of the plant, incubation pot, and other accessories. **Figure 7A** shows that the watering suspension lasts 24 h (four watering events). The plant used three watering events to recover. The abnormal data point was on November 18 due to a minor watering suspension. The abnormal data point on November 22 was due to noises in the raw data. Regardless of the abnormal data, the linear regression yields 5.49 g/day growth with *R* ^2^ = 0.95.

A plant’s water-holding capacity is one of the important plant indices to evaluate plant growth. Plant 38’s dynamic and static water holding capacity are shown in **Figure 7B**. Both the dynamic and static water holding capacity fluctuate daily.

Root water-holding capacity reflects the amount of roots and morphology. Plant 38’s root dynamic water holding capacity data are shown in **Figure 7C**. The daily fluctuations before November 15 were significantly greater than those after November 18. The dynamic water loss (negative mass change) represents the amount of water that exits after the watering event stops. The dynamic water loss slightly decreases (less negative) at 0.24 g/day with *R* ^2^ = 0.78 with abnormal data exclusion.

One of the most important functions of this hydroponic system was to monitor plant growth by evaluating plant weight. As shown in **Figure 7A**, Plant 38’s mass increased linearly as time progressed. Water outages on November 15 and 18 reduced the growth speed. The water outage and recovery also demonstrated the plant’s tolerance, resistance, and response to water stress. The reduced daily fluctuation after the water outage and recovery indicated that the plant was growing conservatively in response to the environmental condition change. Such insights may be useful for commercial hydroponic growers to evaluate emergency water outages and calculate production loss.

As one of the important physiology indices, water holding capacity, shown in **Figure 7B**, signifies the water availability for plants between watering events. The static water holding capacity, $M_{st.h}$ , evaluates the net water retention after water exits. It is the true water amount that plants can use. The dynamic water holding capacity, $M_{dy.h}$ , evaluates the water holding capacity with water running. Beyond static water holding, dynamic water holding also includes the water retention from surface rinsing due to surface tension. There were many factors that can change the surface tension and water retention capacity between the xylem cell wall and watering nutrition liquid, such as temperature, salt concentration (here as electric conductivity), water running speed, xylem cell wall surface roughness, and, most importantly, xylem surface area (Kumar et al., 2022). The daily fluctuations for both $M_{st.h}$ and $M_{dy.h}$ indicate that the water holding capacities are highly correlated with environmental parameters that are cycled every 24 h.

As side evaluation indices, dynamic water loss, $M_{dy.l}$ , and dynamic root holding capacity, $M_{dy.r}$ , shown in **Figure 7C**, provide useful insights. Dynamic water loss is the direct evaluation of the amount wasted after the watering event ends. Reducing this evaluation index would reduce energy usage and improve system efficiency. In this experiment, reduced water loss fluctuations signify that the plant is less susceptible to environmental changes. The dynamic root holding capacity fluctuated less than other evaluation indices from the beginning of the growth progress. $M_{dy.r}$ signifies the power of root growth to prevent water loss. Similar to $M_{dy.h}$ , root surface area significantly impacts $M_{dy.r}$ . Therefore, $M_{dy.r}$ can be treated as the root evaluation index.

### 4.4 Correlation matrices

Plant physiology status is susceptible to environmental condition changes. In this subsection, we use the physiological indices to provide insights into the physiological properties of Plant 38 by computing and plotting the correlation coefficient matrix between the physiological indices and environmental factors and the secondary correlation coefficients matrix between the physiological indices and their temporal variability, which is measured as the moving standard deviation with a step size of four watering events. The full name of the environmental factors and evaluation indices can be found in **Table 1** and Section 3.4

**Figure 8:**
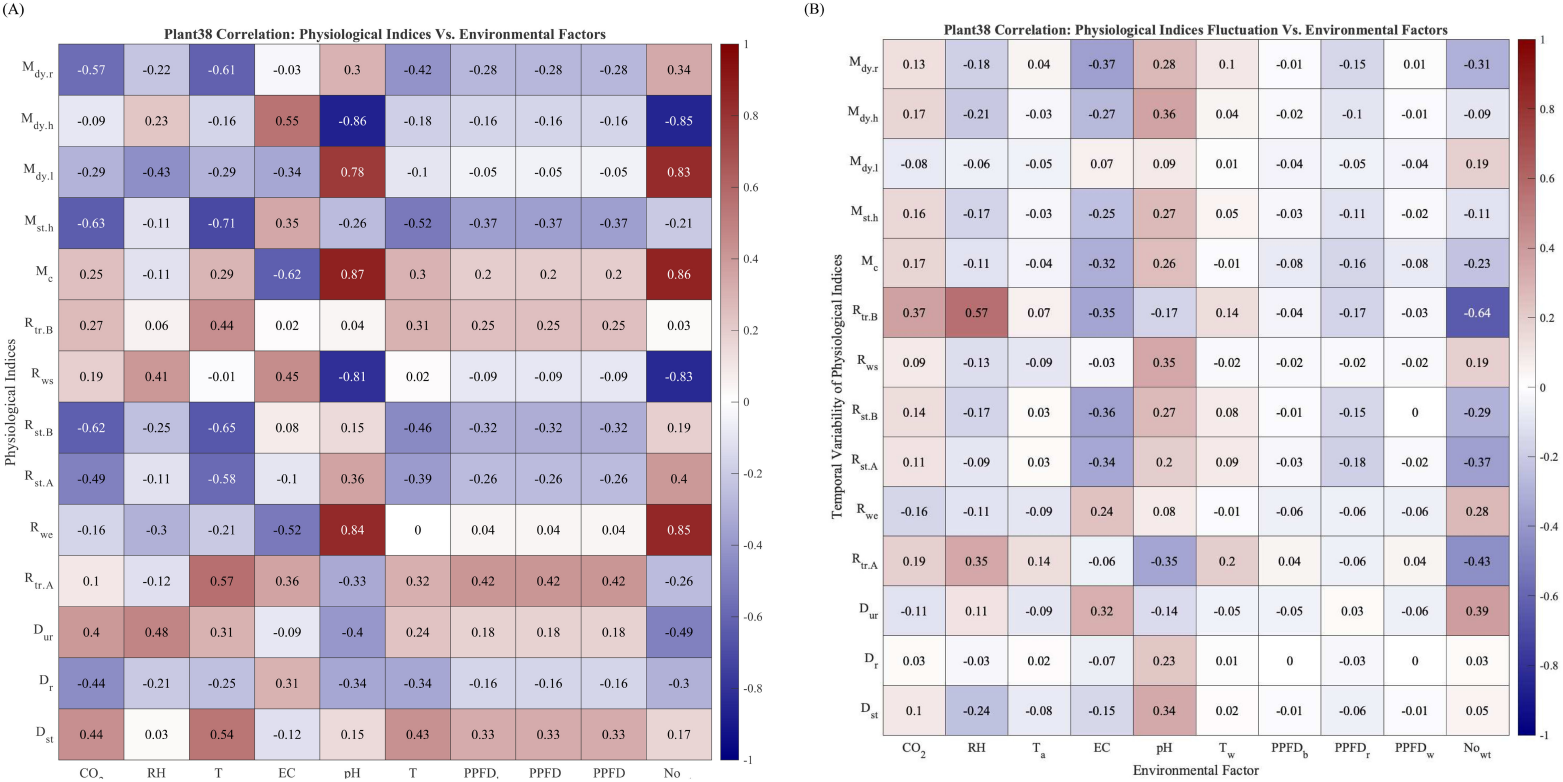
**(A)** Correlation between the physiological indices of Plant 38. No data exclusion. **(B)** Correlation between physiological indices fluctuation Plant 38. No data exclusion.

The correlation matrix heat map of Plant 38 between physiological indices and environmental factors is shown in **Figure 8A**. Several correlation coefficients are worth noting, such as the correlation between pH and $M_{dy.h}\left(–0.86\right)$ , $M_{dy.l}\left(0.78\right)$ , $M_{c}\left(0.87\right)$ , $R_{ws}\left(–0.81\right)$ , and $R_{we}\left(0.84\right)$ . As an important water condition descriptor, pH affects plants’ physiology and water uptake from many perspectives. With lower pH, the higher availability of Ca and Mg ions in the liquid requires plants to uptake higher amounts of water to balance the osmotic pressure between cell membranes. Therefore, the negative correlations between pH and $M_{dy.h}$ and $R_{ws}$ signify a higher water retention at lower pH. The correlation between $No_{wt}$ and $M_{dy.h}\left(–0.85\right)$ , $M_{dy.l}\left(0.83\right)$ , $M_{c}\left(0.86\right)$ , $R_{ws}\left(–0.83\right)$ , and $R_{we}\left(0.85\right)$ .

The correlation matrix between the temporal variability of the physiological index and environmental factors is shown in **Figure 8B**. The correlations between the temporal variability of $R_{tr.B}$ and RH (0.57) and $No_{wt}\left(–0.64\right)$ are also worth noting, meaning that the temporal variability of plant transpiration before watering responds positively to relative humidity and negatively to the number of watering events, $No_{wt}$ . Similar observations have been made in Negrão et al. (2016).

**Figure 9:**
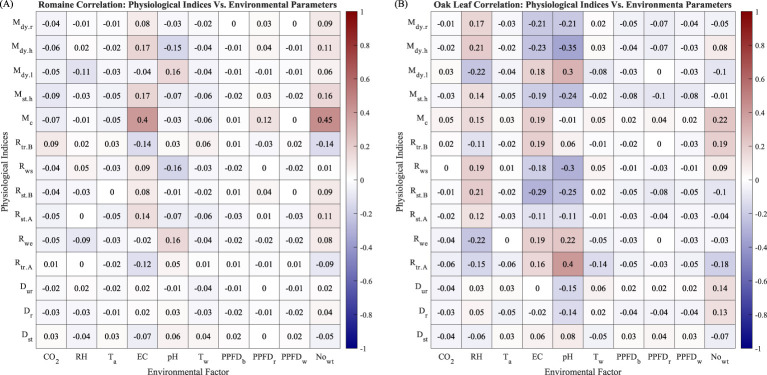
**(A)** Correlation between environmental parameters and evaluation indices of Romaine lettuce. **(B)** Correlation between environmental parameters and evaluation indices of Oak Leaf lettuce.

**Figures 9A, B** are the counterparts of **Figure 8A** for Romaine and Oak Leaf varieties, respectively. The correlation coefficients for $M_{c}$ with EC and $No_{wt}$ for Romaine lettuce in **Figure 9A** are considerably higher than the values in **Figure 9B** for Oak Leaf. The higher correlation coefficient averages in **Figure 9B**, especially for RH, EC, pH, and No.wt suggested that Oak Leaf lettuce is more susceptible to changes of these environmental factors.

## 5 Conclusions

In this study, we developed a non-destructive approach to continuously monitoring hydroponic lettuce’s weight *in situ* with a high-frequency data acquisition system assisted by IoT integration. Such weight data enabled us to define a number of insightful physiological indices to characterize plants’ physiology status, water circulation conditions, and system operation efficiency and to compute the correlation between environmental factors and between these indices and their temporal variability. The physiological properties of different lettuce varieties can also be quantitatively characterized and compared using these correlation matrices.

Our proposed approach is not without its limitations. For example, all the data in the time domain were only analyzed as sample variance. There was no stage segmentation for all evaluation indices. Additional future experiments will be necessary to validate the initial findings of the analyses reported in this paper, such as the different correlation coefficients for the two different lettuce varieties. From the perspective of system hardware, there was hardware to monitor CO_2_, water temperature, pH, and electric conductivity, but not control them precisely. The watering operation parameter was not changed during the entire experiment. Adding such variation may improve the data quality. From the perspective of signal processing and analyzing approaches, the robustness of the signal process code shall be improved to handle irregular weight change profiles. Chlorophyll and fluorescence are also great indicators to evaluate the plants’ physiology. However, they are expensive to measure. As a proof-of-concept study, it is important to develop a cost-effective approach to overcoming the budget constraints.

By providing continuous weight data to other environmental and plant physiological data sets, our study opens doors to numerous future research opportunities. It will be greatly beneficial to define and include new physiological indicators in future research. Another potentially fruitful topic is to use the continuous weight data to propose and validate new hypotheses about the role of water circulation in physiological processes. Numerous models and algorithms could be used for such analysis. For example, Random Forest Variable Importance Analysis could be used to analyze the impact of key environmental factors on plant physiological indicators.

Beyond the current development, the system can be further developed in terms of hardware, software, and implementation. From the hardware perspective, active control of water tank refill, pH control, nutrient ion control, and monitoring can further improve system controllability. More sensors with spatial distribution could improve the system sensing ability of environmental condition changes and, therefore, explore the impact of microenvironmental variation due to crop physiology. From the software perspective, CM could be utilized to further correlate physiology to measurable physics. This system is the foundation of hybrid process-based data-driven CM. It allows training and validating a more accurate and scientifically explainable CM. From the implementation perspective, this system can measure and evaluate other crops, not limited to lettuce.

## Data Availability

The original contributions presented in the study are included in the article/supplementary material. Further inquiries can be directed to the corresponding author.

## References

[B1] Bharti A. Prasanna R. Kumar G. Kumar A. Nain L. (2019). Co-cultivation of cyanobacteria for raising nursery of chrysanthemum using a hydroponic system. J. Appl. Phycology 31, 3625–3635. doi: 10.1007/s10811-019-01830-9

[B2] Chia S. Y. Lim M. W. (2022). A critical review on the influence of humidity for plant growth forecasting. IOP Conf. Series: Materials Sci. Eng. 1257, 12001. doi: 10.1088/1757-899x/1257/1/012001

[B3] Earles J. M. Sperling O. Silva L. C. R. McElrone A. J. Brodersen C. R. North M. P.. (2015). Bark water uptake promotes localized hydraulic recovery in coastal redwood crown. Plant Cell Environ. 39, 320–328. doi: 10.1111/pce.12612 26178179

[B4] Elsallam M. E. A. EL-Moslamy S. H. El-Al A. A. Zahran H. F. (2021). Scaling-up production of cost-effective and eco-friendly bio-fertilizer and its application on barley green fodder via IoT hydroponic system. J. Genet. Eng. Biotechnol. 19, 1–12. doi: 10.1186/s43141-021-00196-1 PMC8239096 34181106

[B5] Gallardo M. Elia A. Thompson R. B. (2020). Decision support systems and models for aiding irrigation and nutrient management of vegetable crops. Agric. Water Manage. 240, 106209. doi: 10.1016/j.agwat.2020.106209

[B6] Gamage D. Thompson M. Sutherland M. Hirotsu N. Makino A. Seneweera S. (2018). New insights into the cellular mechanisms of plant growth at elevated atmospheric carbon dioxide concentrations. Plant Cell Environ. 41, 1233–1246. doi: 10.1111/pce.13206 29611206

[B7] Ibayashi H. Kaneda Y. Imahara J. Oishi N. Kuroda M. Mineno H. (2016). A reliable wireless control system for tomato hydroponics. Sensors 16, 644. doi: 10.3390/s16050644 27164105 PMC4883335

[B8] Kumar A. Bhattacharya T. Mukherjee S. Sarkar B. (2022). A perspective on biochar for repairing damages in the soil–plant system caused by climate change-driven extreme weather events. Biochar 4, 22. doi: 10.1007/s42773-022-00148-z

[B9] Mishra P. Jimmy L. Ogunmola G. A. Phu T. V. Jayanthiladevi A. Latchoumi T. P. (2020). Hydroponics cultivation using real time iot measurement system. J. Physics: Conf. Ser. 1712, 12040. doi: 10.1088/1742-6596/1712/1/012040

[B10] Mittler R. Zandalinas S. I. Fichman Y. Breusegem F. V. (2022). Reactive oxygen species signalling in plant stress responses. Nat. Rev. Mol. Cell Biol. 23, 663–679. doi: 10.1038/s41580-022-00499-2 35760900

[B11] Negrão S. Schmöckel S. M. Tester M. (2016). Evaluating physiological responses of plants to salinity stress. Ann. Bot. 119, 1–11. doi: 10.1093/aob/mcw191 27707746 PMC5218372

[B12] Neocleous D. Savvas D. (2022). Validating a smart nutrient solution replenishment strategy to save water and nutrients in hydroponic crops. Front. Environ. Sci. 10. doi: 10.3389/fenvs.2022.965964

[B13] Ohashi-Kaneko K. Matsuda R. Goto E. Fujiwara K. Kurata K. (2006). Growth of rice plants under red light with or without supplemental blue light. Soil Sci. Plant Nutr. 52, 444–452. doi: 10.1111/j.1747-0765.2006.00063.x 15653806

[B14] Osakabe Y. Osakabe K. Shinozaki K. Tran L.-S. P. (2014). Response of plants to water stress. Front. Plant Sci. 5. doi: 10.3389/fpls.2014.00086 PMC3952189 24659993

[B15] Papanatsiou M. Petersen J. Henderson L. Wang Y. Christie J. M. Blatt M. R. (2019). Optogenetic manipulation of stomatal kinetics improves carbon assimilation, water use, and growth. Science 363, 1456–1459. doi: 10.1126/science.aaw0046 30923223

[B16] Sambo P. Nicoletto C. Giro A. Pii Y. Valentinuzzi F. Mimmo T.. (2019). Hydroponic solutions for soilless production systems: Issues and opportunities in a smart agriculture perspective. Front. Plant Sci. 10. doi: 10.3389/fpls.2019.00923 PMC6668597 31396245

[B17] Samuolienė G. Viršilė A. Miliauskienė J. Haimi P. J. Laužikė K. Brazaitytė A.. (2021). The physiological response of lettuce to red and blue light dynamics over different photoperiods. Front. Plant Sci. 11. doi: 10.3389/fpls.2020.610174 PMC7907654 33643330

[B18] Saraswathi D. Manibharathy P. Gokulnath R. Sureshkumar E. Karthikeyan K. (2018). “Automation of hydroponics green house farming using IOT,” in 2018 IEEE International Conference on System, Computation, Automation and Networking (ICSCA). doi: 10.1109/icscan.2018.8541251

[B19] Sun Y. Frankenberg C. Wood J. D. Schimel D. S. Jung M. Guanter L.. (2017). OCO-2 advances photosynthesis observation from space via solar-induced chlorophyll fluorescence. Science 358, 5747. doi: 10.1126/science.aam5747 29026013

[B20] Thakulla D. Dunn B. Hu B. Goad C. Maness N. (2021). Nutrient solution temperature affects growth and °brix parameters of seventeen lettuce cultivars grown in an NFT hydroponic system. Horticulturae 7, 321. doi: 10.3390/horticulturae7090321

[B21] Woodward I. (2004). Tall storeys. Nature 428, 807–808. doi: 10.1038/428807a 15103357

[B22] Zhang S. Guo Y. Li S. Ke Z. Zhao H. Yang J.. (2022). Investigation on environment monitoring system for a combination of hydroponics and aquaculture in greenhouse. Inf. Process. Agric. 9, 123–134. doi: 10.1016/j.inpa.2021.06.006

